# A Review on the Environmental Fate Models for Predicting the Distribution of Engineered Nanomaterials in Surface Waters

**DOI:** 10.3390/ijms21124554

**Published:** 2020-06-26

**Authors:** Edward Suhendra, Chih-Hua Chang, Wen-Che Hou, Yi-Chin Hsieh

**Affiliations:** Department of Environmental Engineering, National Cheng Kung University, Tainan City 701, Taiwan; edsuhendra@gmail.com (E.S.); whou@mail.ncku.edu.tw (W.-C.H.); likelife3169@gmail.com (Y.-C.H.)

**Keywords:** engineered nanomaterials, environmental fate models, surface waters, ENM fate processes

## Abstract

Exposure assessment is a key component in the risk assessment of engineered nanomaterials (ENMs). While direct and quantitative measurements of ENMs in complex environmental matrices remain challenging, environmental fate models (EFMs) can be used alternatively for estimating ENMs’ distributions in the environment. This review describes and assesses the development and capability of EFMs, focusing on surface waters. Our review finds that current engineered nanomaterial (ENM) exposure models can be largely classified into three types: material flow analysis models (MFAMs), multimedia compartmental models (MCMs), and spatial river/watershed models (SRWMs). MFAMs, which is already used to derive predicted environmental concentrations (PECs), can be used to estimate the releases of ENMs as inputs to EFMs. Both MCMs and SRWMs belong to EFMs. MCMs are spatially and/or temporally averaged models, which describe ENM fate processes as intermedia transfer of well-mixed environmental compartments. SRWMs are spatiotemporally resolved models, which consider the variability in watershed and/or stream hydrology, morphology, and sediment transport of river networks. As the foundation of EFMs, we also review the existing and emerging ENM fate processes and their inclusion in recent EFMs. We find that while ENM fate processes, such as heteroaggregation and dissolution, are commonly included in current EFMs, few models consider photoreaction and sulfidation, evaluation of the relative importance of fate processes, and the fate of weathered/transformed ENMs. We conclude the review by identifying the opportunities and challenges in using EFMs for ENMs.

## 1. Introduction

Engineered nanomaterials (ENMs) are intentionally produced or manufactured materials with at least one dimension in the size range of 1–100 nm [1,2,3]. At this size, materials behave differently to their bulk forms, which is of interest for novel applications [1,2]. Nanotechnology that is the application of scientific knowledge to manipulate and fabricate ENMs and associated products is intensively applied in fields, such as consumer products [4,5,6,7], sporting goods [8], water treatment [9,10,11], photocatalysis [12,13,14], electrocatalysis [15], medicine [16], and renewable energy [17]. ENMs are widely used in consumer products, such as clothes, sox, surface treatment, cosmetics, and packaging [4,5,6,7], potentially contributing to significant ENMs’ release to the environment. For instance, silver nanoparticles (AgNPs) are used in clothes, sox, and packaging due to its antimicrobial properties [18,19]. Titanium dioxide nanoparticles (TiO_2_ NPs) and zinc oxide nanoparticles (ZnO NPs) have applications in surface treatment, e.g., paint and coating, due to its antifouling properties, as well as cosmetics, e.g., sunscreen as ultraviolet (UV)-absorber [5,6,20]. The use of TiO_2_ NPs and ZnO NPs in photocatalysis in the degradation of contaminants, such as •OH, O_2_•-, H_2_O_2_ [12,13], and renewable energy, e.g., H_2_ production [14], is also common. Recent research exists about the applications of nanotechnology in water remediation. For example, due to its ultrahigh surface areas, nanoscale nickel metal organic frameworks (MOFs) decorated over graphene oxide (GO) and carbon nanotubes (CNTs) have been proposed as new adsorbents in water treatment to remove methylene blue (MB) [21], and MOFs-derived magnetic Ni and Cu nanoparticles have been studied in the catalysis of a sodium borohydride (NaBH_4_)-mediated reduction of environmental pollutants, such as 4-nitrophenol, methyl orange, and MB [22]. The upward trend of ENMs’ global production, as well as the widespread applications of nanotechnology could significantly increase the release of ENMs to the environment [23,24].

ENMs are considered as a new class of pollutant due to the unique properties and extraordinary behaviors, which leads to unknown environmental risks [25,26]. While the paradigm used in the risk assessments of regular substances is generally considered to be applicable for ENMs, it is necessary to update the current risk assessment approaches in the evaluation of physicochemical properties, environmental fate and exposure, and (eco)toxicological effects applied for traditional chemicals in regulations in order to be compatible with ENMs’ unique characteristics [27,28,29]. In the past decade, there has been strong progress in the development of methods to evaluate ENMs’ (eco)toxicological effects and bioaccumulation potentials [30,31]. In terms of environmental exposure assessment, this can be done by empirically measuring ENMs in environmental matrices and/or by predicting ENMs’ fate and release in environmental compartments. Despite the considerable progresses made in the detection methods of ENMs, the current analytical technologies could not readily characterize low concentrations of ENMs and distinguish natural nanomaterials from engineered ones in complex environmental matrices [31,32]. Regarding this situation, environmental exposure models could be used as an alternative method to quantitatively predict the environmental distribution of ENMs [28,31,33,34].

Traditionally, exposure models have been used as a tool in the evaluation of environmental concentrations of chemical substances [28,35,36]. Given the recognized properties of ENMs that are distinct from molecular chemicals, it is necessary to review the fate processes of existing exposure models for their relevancy for ENMs and update them accordingly [31,37,38]. For example, the equilibrium partitioning of organic chemicals is not generally considered to be relevant for ENMs [38,39]. The studies on the environmental fate processes of ENMs (e.g., heteroaggregation, dissolution, wastewater treatment removal, and transformation, etc.) have considerably increased in the past one to two decades [31,37]. There is an important opportunity to consider and include these advances in exposure models to allow for improved prediction of ENMs’ environmental distribution.

Prior reviews on the environmental fate and exposure modeling of ENMs exist. Dale et al. [33] reviewed the nano-specific processes that can be included in engineered nanomaterial (ENM) fate models of aquatic systems. The review of Baalousha et al. [40] discussed the modeling of ENM fate and biological uptake in aquatic and terrestrial environments and also emphasized the considerations of nano-specific processes. Nowack [28] reviewed environmental exposure models for ENMs in a regulatory context with a large focus on the material flow analysis models (MFAMs). He concluded that current MFAMs and environmental fate models (EFMs) are both generally compatible with the framework of exposure assessment accepted in regulatory and that both models require proper validation. William et al. [34] reviewed the nano-specific processes as well as the releases, forms, and particle sizes of ENMs considered in recent aquatic fate models. Since then, significant progresses in ENM fate modeling in the aquatic environment have been made, especially in surface waters, that provide good spatial and temporal resolution with the consideration of new nano-specific processes, such as sunlight-driven photoreactions; however, these recent advances have not generally been included in existing reviews [41,42].

The potential of ENMs released to groundwater exists from groundwater remediation using nano zero-valent iron (nZVI) [43]. While there are few nZVI groundwater modeling studies, these current studies are limited to nZVI rather than other ENMs [44,45,46]. As such, this review does not focus on groundwater and this could be a direction for future reviews when more related work emerges.

The purpose of this paper is to comprehensively review the recent advances in the environmental exposure assessment of ENMs using computational approaches. While ENM exposure models in other environmental media, such as soil and air, are also covered, we focus on the latest advances of surface water models.

We also comprehensively review recent studies on the environmental fate processes of ENMs to identify emerging processes that can be included in the fate models towards improved prediction of ENMs’ distribution in environmental matrices. Current exposure models of ENMs may be largely classified into two major types, which are MFAMs and EFMs [28,33,40]. MFAMs quantify the release of ENMs in life-cycle stages from production to waste disposal/recycling into the environment [47,48,49,50,51], while EFMs consider the mechanistic fate and transport processes (i.e., nano-specific fate processes) in the prediction of ENMs’ concentrations in or across environmental compartments (e.g., water, sediment, soil, or air) [52,53]. EFMs could be further classified to multimedia compartmental models (MCMs) [36,54,55,56,57] and spatial river/watershed models (SRWMs) [35,41,42,58,59]. We discuss and compare the functions and utilities of recent ENM exposure models. We conclude the review by identifying the opportunities and challenges of using EFMs for predicting the environmental exposure of ENMs.

## 2. Exposure Models for the Prediction of Engineered Nanomaterials’ (ENMs’) Concentrations in Surface Waters

Obtaining the temporal–spatial distribution information of ENMs in a water environment is the basis of ENMs’ exposure and risk assessment. For example, there is no potential risk of a chemical substance on the ecosystem if the predicted levels of ENMs in the environment (e.g., predicted environmental concentrations, PECs) are lower than the predicted no effect concentrations (PNECs) [36,47,60]. Before the direct and quantitative measurement methods of ENMs in natural systems are developed, current information of PECs for risk assessment is mainly estimated by exposure models. The exposure models that have been intensively applied in conventional chemical risk assessments are starting to be used for ENMs with modifications [28,33,34,40].

In the last decade, several types of exposure models have been applied to estimate environmental releases and concentrations of ENMs. These models can be classified to MFAMs that predict releases from production, fate in technical systems, and ultimate releases to the environment, and EFMs that predict ENMs’ concentrations by including ENM fate processes as well as the distribution within environmental compartments [28]. The recent development of ENM exposure models are described as follows.

### 2.1. Material Flow Analysis Models (MFAMs)

Material flow analysis (MFA) is a mass balance-based system approach, which tracks and quantifies inventories and flows of materials throughout the entire life cycle in a well-defined geographic boundary or time span that is covered by the system. Many nanomaterial models (e.g. MFAMs) rely on MFA to predict releases from products, fate (i.e., removal) in technical systems, and final releases to the environment [28]. The outputs provided by MFAMs can be linked to EFMs as system input data. For example, the more realistic ENMs’ release information resulting from MFAMs, e.g., the released forms and particle size distributions of ENMs, can be used by EFMs as a basis for choosing relevant environmental fate processes and hence allowing EFMs to better quantify the transport and distribution between and within environmental compartments. Generally, MFAMs are only ENM release models, although some extended MFAMs are also simplified EFMs, which estimate environmental concentrations by assuming standard sizes of environmental compartments and complete mixing [28]. The current MFAMs of ENMs are shown in Table 1 with the following review.

Mueller and Nowack [47] used an MFA approach for quantifying the releases of AgNPs, TiO_2_ NPs, and CNTs from products, and predict their expected concentrations in soil, air, and water compartments of Switzerland. The resulting environmental concentrations from MFAMs are mainly controlled by how the system was defined, e.g., boundary and time span, as well as how the ENM specific input parameters were determined, such as the estimated production volume, the allocation of the production volume to product categories, the particle discharge from products, and the transfer coefficients between environmental compartments. As a significant amount of input information was estimated, not available, or have uncertainty, the system was usually assumed in a steady state, ENMs were released completely from products in one year, and environmental compartments were considered to be homogeneous and well mixed [47].

Probabilistic material flow analysis models (P-MFAMs) are then used with the employment of probability distributions for model input and/or output to address the inconsistency and variability of model input parameters, as well as to model PECs for ENMs, such as TiO_2_ NPs, ZnO NPs, AgNPs, CNTs, and fullerenes [48,49,61,62].

Dynamic probabilistic MFAMs (DP-MFAMs) address the time-dynamic behavior of the system for material flows over several consecutive periods, considering changes in the inflow to the system and intermediate delays in local stocks. Unlike the static MFA, the dynamic MFA considers a reasonable time frame of a period rather than a single year and time-dependent ENMs’ release from products over the life-cycle of the products rather than the simple assumption that ENMs are released completely from all applications in the single production year [50,51,63].

**Table 1:** Review of existing environmental exposure models for engineered nanomaterials (ENMs).

Model Classification	Model Name	Model Features	Compartments Considered	Fate Processes	References
Material flow analysis models	MFAMs	Steady state, less information required, simplified structure	Air, water, soil	-	Mueller and Nowack (2008) [47]
	P-MFAMs	Accounting for the uncertainty of model input parameters using probabilistic distribution	Air, water, soil, sediment	-	Gotschalk et al. (2009) [61], Gotschalk et al. (2010) [48], Gotschalk et al. (2011) [62], Sun et al. (2015) [49], Liu et al. (2015) [56]
	DP-MFAMs	Accounting for time-dependent changes in the system behavior	Air, water, soil, sediment	-	Bornhöft et al. (2016) [50], Sun et al. (2016) [63], Wang and Nowack (2018) [51]
Multimedia compartmental models	MendNano	Intermedia transport processes included partitioning ratios	Air, water, soil, sediment, biota	Homoaggregation, heteroaggregation, dissolution	Liu and Cohen (2014) [54]
	SimpleBox4 Nano (SB4N)	Steady state environmental ENM fate processes are modeled mechanistically using first-order rate constants	Air, water, soil, sediment	Heteroaggregation, dissolution	Meesters et al. (2014) [36]
	RedNano	A model system which combines a P-MFAMs based release model (LearNano) and a multimedia fate model (MendNano)	Air, water, soil, sediment, biota	Homoaggregation, heteroaggregation, dissolution	Liu et al. (2015) [56]
	SimpleBox4 Nano (SB4N)	Steady state environmental ENMs’ fate, probabilistic distribution	Air, water, soil, sediment	Heteroaggregation, dissolution	Meesters et al. (2016) [55]
	nanoFate	Dynamic environmental ENMs’ fate	Air, water, soil, sediment	Heteroaggregation, dissolution	Garner et al. (2017) [57]
Spatial river/watershed models	Rhine river box model	Steady state box model	Water, sediment	Heteroaggregation	Praetorius et al. (2012) [35]
	Rhone river box model	Cluster analysis, steady state box model	Water, sediment	Heteroaggregation	Sani-Kast et al. (2015) [64]
	Diagenesis model	1-D sediment diagenesis model	Freshwater sediment	Dissolution, sulfidation	Dale et al. (2013) [65]
	GWAVA	Gridded probability distribution	Water	Dissolution	Dumont et al. (2015) [66]
	Nano DUFLOW	1-D unsteady flow in open-channel systems	Water, sediment	Homoaggregation, heteroaggregation, dissolution	Quik et al. (2015) [58], Klein et al. (2016) [67]
	SOBEK river-DELWAQ	A model system integrates open channel hydraulics and water quality models	Water	Homoaggregation, heteroaggregation, dissolution	Markus et al. (2016) [68]
	WASP7–HSPF	Dynamic, mass-balance, spatially resolved differential fate and transport modeling framework	Water, sediment	Dissolution, sulfidation	Dale et al. (2015) [59]
	WASP8	A detailed surface water quality model with ENM fate and transport processes	Water, sediment	Dissolution, sulfidation, heteroaggregation, photoreaction	Bouchard et al. (2017) [41], Han et al. (2019) [42]
	SWMM-EFDC	Suitable for urban stormwater and sewage systems; coupling both surface hydrology and hydrodynamic models	Water, sediment	Heteroaggregation, dissolution	Saharia et al. (2019) [69]

### 2.2. Environmental Fate Models (EFMs)

EFMs are exposure models, which apply a quantitative and mechanistic description of fate processes to predict the behavior as well as the concentration of the contaminant in the environment. The basic concept governing of EFMs is the integration of pollutant transport, transfer, and degradation processes into mass balance equations for the pollutant. EFMs consist of mass balance equations for a chemical in a system of coupled environmental compartments in which the coefficients are generally based on extensive empirical data [52,53].

EFMs of ENMs can be classified into MCMs and SRWMs. MCMs comprise the mechanistic description of fate and transport processes of ENMs as intermedia transfer incorporating well-mixed environmental compartments (e.g., air, water, soil, and sediment) while SRWMs are dynamic and spatially resolved models, which incorporate variation in stream hydrology and morphology as well as sediment transport in river networks, and have the capability to consider the description of water quality linked to the watershed hydrology [28]. The current EFMs of ENMs (MCMs and SRWMs) are shown in Table 1 with the review as follows.

#### 2.2.1. Multimedia Compartmental Models (MCMs)

MCMs treat various environmental media (e.g., surface water, groundwater, and atmosphere) as an integrated system, synthesizing information about chemical partitioning, transformation, and intermedia transport. The models have been used to predict the distribution of the contaminant based on mass balance equations at a regional and global scale. The environment is described as a set of well-mixed compartments, with each acting as a particular medium (e.g., air, water, and soil). The model consists of the compartmental mass balance ordinary differential equations (ODEs), which can be solved either at steady state or dynamically with the time steps selected [52,53,54]. The existing multimedia compartmental models are summarized in Table 1 with the review as follows.

MendNano was developed by Liu and Cohen [54] to predict environmental exposure concentrations of ENMs dynamically. The model consists of the compartmental mass balance ODEs, which can be solved with the time steps selected, with the consideration of ENM fate processes such as homoaggregation, heteroaggregation, and dissolution as intermedia transport between bordering compartments. For example, attachment factors are used in homoaggregation and heteroaggregation, which are defined as fractions of ENMs attached to themselves or ambient particles [54]. Later, RedNano is developed as an improvement of MendNano by coupling the model with LearNano as P-MFAMs in order to derive more realistic ENMs’ release rates [56].

Then, SimpleBox4Nano (SB4N), which is the modification of a classical multimedia mass balance model in European Regulation on Registration, Evaluation, Authorization, and Restriction of Chemicals (REACH) called the SimpleBox model, was introduced by Meesters et al. [36]. It is solved at steady state with the consideration of ENM fate processes such as heteroaggregation and dissolution. Unlike MendNano, SB4N used first order rate constants for ENM fate processes, with the exception of dissolution, which was considered as a loss mechanism. Heteroaggregation is modeled between ENMs with natural colloids (< 450 nm) and larger suspended particles (> 450 nm). The ENMs’ input is only free dispersive ENMs. However, the output has three ENMs’ states as a result of heteroaggregation, which are: (1) Freely dispersed ENMs; (2) ENMs attached with natural colloids; and (3) ENMs heteroaggregated to larger suspended particles [36]. Subsequently, Meesters et al. [55] analyzed the sensitivity of SB4N to uncertainties in emission estimations; physicochemical properties of cerium oxide nanoparticles (CeO_2_ NPs), ZnO NPs, and TiO_2_ NPs; and natural variability of environmental systems by incorporating Monte Carlo simulations as a probabilistic approach.

Recently, Garner et al. [57] proposed a multimedia dynamic model called nanoFate, which incorporates a broader range of ENM processes (e.g., emissions from their manufacturing, use, and disposal) and climate variability (e.g., the use of observed daily hydrometeorological data) with the consideration of heteroaggregation and dissolution using first rate order rate constants. NanoFate can also quantify the concentration of dissolved ions as a result of the dissolution process. Furthermore, nanoFate considers not only the point source but also the non-point source, such as ENMs’ loading from the use of biosolids in agriculture. Runoff was calculated using the runoff equation as well as soil loss resulting from erosion, and a variety of land uses were considered.

#### 2.2.2. Spatial River/Watershed Models (SRWMs)

Spatial river models are dynamic and spatially resolved models that consider variability in stream hydrology and morphology as well as sediment transport in river networks. Water quality functions of these models are provided by linking hydrologic and water quality models. Hydrologic and water quality models can be either integrated as one model or separated into hydrologic models and water quality models. Watershed models underline the description of watershed hydrology and water quality, including runoff, erosion, and wash off of sediments and pollutants. When coupled to hydrologic and water quality models, watershed models offer more realistic input parameters for hydrology as well as sediment and pollutant loading [59,70]. The current SRWMs are shown in Table 1 with the review as follows.

Praetorius et al. [35] modified the river box model for ENMs in Rhine river based on established multimedia box models for organic chemicals with the inclusion of nano-specific process descriptions. The model framework is similar to MCMs, which can be solved at steady state or as a function of time, but with the consideration of spatial resolution (e.g., Rhine river is divided into 520 “boxes” representing river sections). Heteroaggregation and dissolution are considered as ENM fate processes, with the assumption of attachment efficiency of heteroaggregation in several scenarios [35]. Sani-Kast et al. [64] improved this river box model by incorporating spatial variability in environmental conditions for the Rhone river with cluster analysis.

Subsequently, Dale et al. [65] applied a simple 1-D diagenetic model for predicting AgNPs‘ distribution and silver speciation of resulting Ag ions in freshwater sediments. The model is a modification of a mass balance model by Di Toro et al. [71] that quantifies the speciation of cadmium in sediments considering the sediment as a function of the oxygen depletion in the time of mineralization or organic carbon diagenesis. The model considered the sulfidation and oxidative dissolution process of AgNPs as a function of dissolved oxygen, sulfide, and temperature. The model was then calibrated to data collected from AgNPs-dosed large-scale freshwater wetland mesocosms, and estimated ion release as a result of AgNPs’ sulfidation [65].

DUFLOW simulates 1-D unsteady flow in open-channel systems with the calculation of water levels and flow rates based on the St. Venant equations of continuity and momentum using initial and boundary conditions. NanoDUFLOW was developed by Quik et al. [58] as a modification of the DUFLOW hydrology model, which allows the inclusion of ENM fate process descriptions such as homoaggregation, heteroaggregation, and dissolution to a spatially explicit hydrological model. The model was toward validation, which compared measured and modeled concentrations of < 450 nm cerium (Ce), aluminum (Al), titanium (Ti), and zirconium (Zr)-based particles for the Dommel river, Netherlands [67].

The Global Water Availability Assessment Model (GWAVA) is a catchment gridded hydrology model, which was applied to simulate the spatial distributions of PECs by assigning distinguished ENM loading inputs into each grid [66]. ENM fate processes in GWAVA such as heteroaggregation and dissolution are computed by first-order sink processes.

With the SOBEK-River hydrology model for the simulation of water flows and morphological changes in open channel systems, and the DELWAQ for water quality modelling in 1-D, 2-D, or 3-D surface water systems, Markus et al. [68] developed the SOBEK-River-DELWAQ model system for estimating the temporally and spatially varying ZnO NPs concentration as different ENMs’ states (free, homoaggregates, and heteroaggregates) in Rhine river. The model was then validated by the comparison between the simulation of the total concentration of zinc over time to the measured total concentration of zinc for the Rhine river.

Dale et al. [59] used a dynamic water quality model Water Quality Analysis Simulation Program (WASP) to simulate ZnO NPs and AgNPs and their transformation byproducts in James River Basin, Virginia. WASP version 7 (WASP7) can be coupled with a watershed model to simulate ENMs’ daily stream loads from effluent discharges. WASP is a dynamic generalized modeling framework based on the finite-volume concept to quantify the fate and transport of water quality variables in surface waters, including toxic substances in 1-D, 2-D, or 3-D. WASP7 considers transformation processes, such as dissolution and sulfidation; however, it excludes ENM fate processes, such as heteroaggregation. Hydrology Simulation Program Fortran (HSPF) is an integrated basin-scale model that combines watershed processes with in-stream fate and transport in one-dimensional stream channels. WASP7 was coupled with Chesapeake Bay Watershed Model (WSM) as an adaption of HSPF to simulate AgNPs and ZnO NPs as well as the total concentration of Ag^+^ and Zn^2+^ as their speciation in James River Basin, Virginia.

WASP7 was updated to WASP version 8 (WASP8) with the inclusion of ENM fate processes, such as heteroaggregation and photoreaction. In WASP8, ENM fate processes can be parametrized using experiment-based kinetics. Heteroaggregation is described as the attachment efficiency (α_het_). Bouchard et al. [41] used WASP8 to simulate multiwalled carbon nanotubes (MWCNTs) fate and transport in Brier Creek. In this study, α_het_ was derived from lab-scale experiments by using natural surface water with a range of ionic strengths. The phototransformation rate constant is calculated using the average light intensity in the surface water, which depends on light attenuation. Light attenuation is calculated for each wavelength band of UV and visible light. Han et al. [42] applied WASP8 to model predicted environmental concentrations of GO and its major reaction phototransformation product reduced GO (rGO) in Brier Creek, GA.

In a recent work, Saharia et al. [69] integrated an urban hydrologic-hydraulic model (Storm Water Management Model, SWMM), and a 3-D hydrodynamic and water quality model (Environmental Fluid Dynamics Code, EFDC) to estimate TiO_2_ NPs concentrations in combined sewer overflows and receiving rivers. SWMM is a dynamic rainfall-runoff simulation model applied to urban areas. EFDC is a three-dimensional modeling system having hydrodynamic, water quality-eutrophication, sediment transport, and toxic contaminant transport components. The modeling results show that heteroaggregation and sedimentation are the key fate processes of TiO_2_ NPs, while dissolution is almost negligible.

## 3. Engineered Nanomaterial (ENM) Fate Processes in Surface Waters

**Figure 1:**
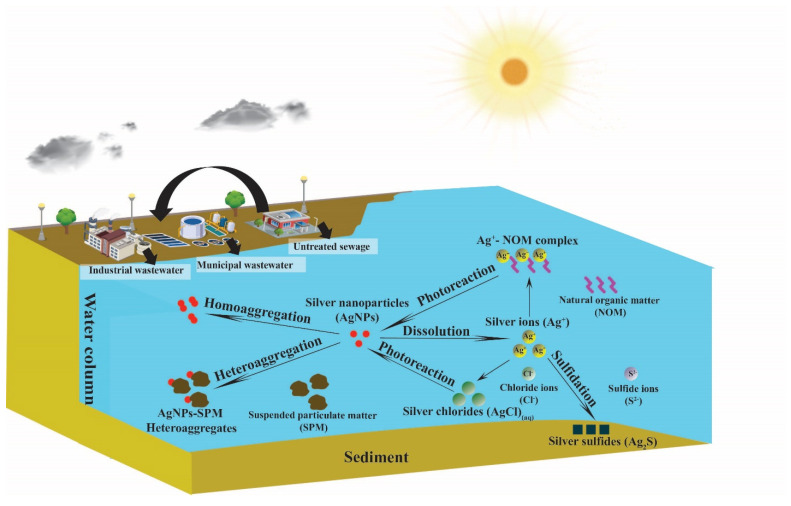
Engineered nanomaterial (ENM) fate processes in surface waters.

The understanding of ENMs’ environmental behaviors is fundamentally important in establishing fate models for ENMs in the environment. In this section, we review the state of the art in the studies of ENM fate processes in the aquatic environment. Possible fate processes of ENMs are usually first investigated in laboratory experiments using simulated environmental conditions to evaluate their potential to occur in natural systems. The current literature suggests that ENMs, depending on specific types, can undergo physical transformation processes, including aggregation (homoaggregation and heteroaggregation), as well as chemical transformation processes, including dissolution, sulphidation, and photoreactions [31,37,72,73]. Figure 1 shows the environmental fate processes of ENMs in surface waters. Here, AgNPs are used as an example to illustrate the ENM fate processes because AgNPs have a wide variety of fate processes that are also common to other ENMs, such as aggregation (homoaggregation and heteroaggregation), dissolution, and sulphidation, and/or specific to themselves, such as photoreactions. These ENM fate processes are discussed as follows.

### 3.1. Aggregation

Aggregation is a generally recognized ENM fate process that is controlled by particle–particle interactions where particles collide and stick to each other to form larger clusters [74,75]. The classical Derjaguin–Landau–Verwey–Overbeek (DLVO) theory describes the tendency of colloids to aggregate, a process that is controlled by the combination of attractive and repulsive forces between charged colloid surfaces in a liquid medium [74]. Currently, this DLVO theory is being improved for diverse complexity, such as the electric double layer interaction energies, polyelectrolyte coating, effects of uncharged polymeric coating, and elastic steric stabilization on the van der Waals force [75]. The application of this theory to ENMs overall works well in helping to understand charge destabilization. The attachment efficiency α (0 ≤ α ≤1) that determines if the collision between particles leads to the attachment of particles (i.e., forming aggregates) is usually used to model the aggregation kinetics of ENMs and is used in ENM fate models [35,36,41,75]. Figure 1 shows that two forms of aggregation may occur: homoaggregation among the same ENMs, or heteroaggregation among ENMs and other particles [37,76,77,78]. Due to the complexity of heteroaggregation, the appropriate method to derive the attachment efficiency of heteroaggregation (α_het_) is still challenging [38,79,80]. Nevertheless, experimental parameterization could be a promising method in determining α_het_ [41,42,80].

Due to the larger concentrations of environmental particles (e.g., suspended particulate matters (SPMs)) compared to ENMs, heteroaggregation with natural particles has been suggested to play a key role in ENM aggregation in the aquatic environment compared to homoaggregation [31,37]. Greater cluster sizes due to aggregation cause increasing settling velocity, thereby more quickly removing ENMs as heteroaggregates from environmental compartments (air, water) into soil or sediments [77,78]. Heteroaggregation also tends to affect the bioavailability and toxicity of ENMs [81,82], for example, heteroaggregation of GO and aluminum oxide (Al_2_O_3_) particles could diminish the bioavailability and toxicity of GO to freshwater algae [81]. As a result, heteroaggregation of ENMs are predominantly considered in recent EFMs compared to homoaggregation as indicated by Table 1.

### 3.2. Dissolution

Dissolution of ENMs can be considered as chemical transformation processes of ENMs from particulate forms into dissolved ions as shown in Figure 1. Dissolution is particularly relevant for metallic and metal oxide ENMs, such as AgNPs, ZnO NPs, and copper oxide nanoparticles (CuO NPs), which have been shown to dissolve to Ag^+^, Zn^2+^, and Cu^2+^, respectively, in aqueous solutions [83,84,85,86,87]. In contrast, metal oxide nanoparticles, such as TiO_2_ NPs, CeO_2_ NPs, and silicon dioxide nanoparticles (SiO_2_ NPs), are fairly insoluble in environmental waters [31]. Water chemistry factors also affect the dissolution of ENMs. Generally, decreased pH increases the dissolution of metallic and metal oxide ENMs [83,84,85,86,87]. Additionally, agglomeration of ENMs tends to diminish the dissolution rate by reducing the diffusion of species involved in dissolution reactions [82,87]. On the other hand, the presence of natural organic matters (NOMs), which are ubiquitous in surface waters, could increase dissolution by enhancing metal solubility through complexation with dissolved metal ions from ENMs [87]. For carbon-based ENMs, dissolution and aqueous solubility are generally not applicable, although fullerene C_60′_s hypothetical aqueous solubility has been reported at a fairly low concentration of 7.96 ng/L [88,89]. Dissolution could be important in the fate and toxicity for ENMs, such as AgNPs. Generally, dissolved ions exert a greater impact to living creatures compared to their particulate forms. Therefore, for some metallic and metal oxide ENMs, dissolution correlates with increased toxicity [31,37]. The important role of dissolution in ENMs’ fate and toxicity has been reflected in its increased inclusion in current exposure models. For example, the dissolution of AgNPs, ZnO NPs, and CuO NPs has been considered in recent fate models as shown in Table 1.

### 3.3. Sulfidation

Sulfidation is a predominant transformation process for some metal or metal oxide ENMs, such as ZnO NPs and AgNPs, especially in an environment with high sulfide concentrations, such as in wastewater treatment plants (WWTPs) or other anaerobic environment [90,91,92,93] as described in Figure 1. The reaction mechanism of sulfidation requires both sulfide and dissolved oxygen and may be either a fast direct reaction or a slower indirect reaction, which could produce metal sulfide ENMs [94]. The low solubility of metal sulfide ENMs, such as silver sulfide nanoparticles (Ag_2_S-NPs), mostly reduce their toxicity in the short term [95,96]; however, a recent study by He et al. [96] has shown transformation of Ag_2_S-NPs potentially resulting in Ag^+^ and in situ formation of Ag^0^ and Ag^0^/Ag_2_S-NPs hetero-nanostructures, which may enhance the toxicity in the long term. Sulfidation may impact metallic ENMs’ toxicity by altering their physicochemical properties. For instance, ZnO NPs’ sulfidation does not form a protective shell of zinc sulfide (ZnS) on the ZnO core or impact its dissolution rate; however, AgNPs’ sulfidation forms a passivating shell of silver sulfide (Ag_2_S), which makes partially sulfidized AgNPs exhibit a far lower solubility than untransformed AgNPs [59]. Generally, the high insolubility of sulfide form, e.g., the formed Ag_2_S shell, could reduce AgNPs’ toxicity compared to the pristine one. However, the sulfidation process is not commonly included in current EFMs. Two studies conducted by Dale et al., using a simple diagenesis model to quantify AgNPs’ sulfidation in freshwater sediments [65], and using WASP7 for modeling sulfidation of AgNPs and ZnO NPs in the rivers [59].

### 3.4. Photoreaction

Photoreaction can be considered as an emerging fate process in the modeling of ENMs’ fate. In a 2019 study, Han et al. [42] first considered sunlight-driven phototransformation in modeling the fate and transport of GO in surface water. While the incorporation of ENM photoreaction in fate modeling is relatively new, there have been many existing process studies on ENM photoreaction. We and other groups have reported on the phototransformation of fullerenes C_60_ and C_70_ [97,98,99,100], CNTs [101,102,103], GO [104,105], AgNPs [72,73,106], and metal dichalcogenides [107]. Photoreaction of ENMs is important, because the process is driven by environmentally relevant sunlight; therefore, it plays a role in the transformation fate of ENMs in surface waters as shown in Figure 1.

Photoreactions under sunlight conditions can result in the oxidation (i.e., photo-oxidation) and/or reduction (i.e., photo-reduction) of ENMs and/or their products and cause structural alterations or degradation of ENMs. For example, Hou et al. ([97,108]) found that photo-oxidation of C_60_ aggregates (nC_60_) results in polyhydroxylated C_60_ photoproducts’ formation driven by nC_60′_s strong light absorption within the solar spectrum. Another example is GO, which undergoes rapid phototransformation under sunlight conditions, resulting in more persistent rGO-like photoproducts. It has been shown that rGO photoproducts exhibit transport and toxic properties unique from pristine GO [105,109,110]. Therefore, it is also important to be able to model the fate of transformed ENMs, due to their fate and biological properties distinct from pristine materials.

There has been a plethora of studies regarding ENM-enabled photocatalysis in the degradation/transformation of pollutants (e.g., water/wastewater treatment) [12,13,111] or in the production of valuable chemicals (e.g., H_2_) [14]. ENMs, such as TiO_2_ NPs and ZnO NPs, are probably the most widely studied photocatalysts [12,13]. Photocatalysis depends on the photocatalyst’s capability to absorb light, create electron–hole pairs, and generate a range of reactive oxygen species (ROS), such as •OH, O_2_^•-^, H_2_O_2_, etc., able to undergo further redox reactions that ultimately degrade/transform pollutants [12,13,111]. Regardless, these results may not be directly applicable to evaluate the photochemical fate of ENMs in the aqueous environment. For example, photocatalytic studies mainly concern the degradation of pollutants or the product formation (e.g., H_2_) [12,13,14,111], with less emphasis on the physicochemical transformation and associated kinetics of the nano-catalysts under environmentally relevant sunlight conditions, which is the information needed in evaluating the photochemical fates of ENMs in the environment. 

## 4. Path Forward 

Based on the review of recent advances in environmental exposure models for ENMs and studies of emerging ENM fate processes, in this section, we discuss and identify the opportunities and challenges in using exposure modeling for risk evaluation of ENMs. The opportunities are related to the consideration of emerging ENM fate processes in current and future EFMs, the evaluation of the relative importance of ENM fate and transport processes within EFMs, modeling the fate and transport of weathered/transformed ENMs, and realistic parametric input of ENM fate processes. The challenge is related to the model validation. We also discuss the model applicability in regulatory context.

This review indicates that studies on the emerging ENM fate processes, such as photoreaction and sulfidation, that are relevant for ENMs, such as AgNPs, carbon-based ENMs, and ZnO [72,73,91,92,93,94,96,97,100,102,103,104,105], have grown significantly in the past 5–10 years, but these processes are not commonly included in recent EFMs [42,59,65]. For most EFMs, either MCMs or SRWMs, the most common ENM fate processes considered have been heteroaggregation and dissolution [36,41,54,57,59,68,69]. In the future, as the studies on emerging ENM fate processes continue to increase, there is a valuable opportunity to consider and evaluate them in EFMs. This would also require the translations of ENM fate process studies that are usually conducted in the laboratory under simulated environmental conditions into mathematical expressions and computer codes.

The addition of emerging ENM fate processes into EFMs needs to be accompanied by the evaluation of their relative importance to the more commonly included processes, such as heteroaggregation, dissolution, and others. The specific processes considered are also dependent on the environmental compartments (e.g., surface water columns, anaerobic sediments, etc.) to be modeled. Recent studies have begun to consider this aspect. For example, Meesters et al. [55] analyzed SB4N’s sensitivity using probability distributions to environmental concentrations, distributions, and speciations of TiO_2_ NPs, CeO_2_ NPs, and ZnO NPs in Europe, with the results showing that heteroaggregation is the dominant process for TiO_2_ NPs and CeO_2_ NPs as well as dissolution for ZnO NPs. A similar approach was also used to determine the most crucial physicochemical properties governing the environmental fate and transport of ENMs in this study. Transformation rate constants and attachment efficiency are found to be the most sensitive physicochemical properties, which can lead to the dominant ENM fate processes governing the environmental fate and transport of ENMs [112]. Future research could have similar considerations that are expected to result in a better understanding of the relative importance of ENM fate and transport processes, particularly when new processes are being evaluated. The relative importance of the ENM fate processes may not always be easy to be measured quantitatively in simulated experiments or in the field, as they often occur simultaneously.

It has been widely shown that some ENMs can transform physically or chemically into products or species that have distinct environmental and ecotoxicological properties from their parent materials [37]. This would include ENMs that are weathered and released from products, such as polymeric nanocomposites, from which released ENMs are often embedded in polymer matrices [113,114,115,116]. Some have argued that transformed ENMs are most environmentally relevant [31,37]. Therefore, modeling the fate and transport of transformed ENMs is equally, if not more, important. Recent research has begun to model the fate and transport of transformed ENMs, rather than just considering transformation as a sink process. For instance, the fate of dissolved products of ZnO NPs and CuO NPs (i.e, Zn^2+^ and Cu^2+^) has been modeled in San Francisco Bay [57]. Other studies modeled the fate and transport of ions (Ag^+^ and Zn^2+^), Ag_2_S, and rGO as the dissolved, sulphidation, and phototransformation products of AgNPs, ZnO NPs, and GO in river systems [42,59]. Further research could continue to model these existing transformed ENMs and also consider other less common ones, such as ENMs released from industrial or consumer products that are embedded in product matrices (e.g., carbon nanotubes embedded in polymers).

Using realistic parametric inputs/estimations to model ENM fate and transport processes creates opportunities to improve the accuracy of EFMs, particularly for site-specific models, such as SRWMs. ENM fate process parameters used in EFMs can be assumed, obtained from the literature, or from experiments. Extensive process studies have shown that environmental factors, such as the ionic strength, particulate, and NOMs, can affect the heteroaggregation and dissolution of ENMs [31,79,87]. These factors often vary depending on the specific sites [117]. Modeling work in the early development of EFMs used assumed parameters, such as attachment efficiencies, given that the information about ENM fate parameters was limited [35], so these parameters are assumed as necessary. Some model studies also extracted ENM fate parameters from the literature [36,54,58,59]. Recent EFMs started to use experimentally derived fate parameters as inputs to models. For instance, studies based on WASP8 derived the attachment efficiency (α_het_) for heteroaggregation processes using laboratory experiments where water and sediment samples collected from the specific sites (i.e., site to be modeled) were mixed with CNTs and GO [41,42]. Experimental parameterization could result in more accurate ENM fate parameters, reflective of specific sites [80]. It is noted that while the experimental derivation of fate parameters can be used for local scale models, it may not be applicable to larger regional or global-scale models where the fate behaviors are usually averaged.

All models require appropriate validation, but this remains a challenge for many current ENM exposure models. The validation of PECs derived from exposure models remains limited due to the lack of suitable analytical methods for ENMs’ measurement in complex environmental matrices [28,40]. Nevertheless, there are recent efforts in the validation of other model outcomes (e.g., dissolved ions) or input parameters in several spatial river/watershed models. For example, Quik et al. [58,67] tried to validate the NanoDUFLOW model by comparing the concentrations of ENMs predicted by the model and the measured concentrations of particles with sizes < 450 nm. The measurement concentrations of < 450 nm size particles are deliberate as an important step in the ENMs’ validation of EFMs [58,67]. Another study validated the SOBEK river-DELWAQ by comparing the simulated total zinc concentration over time to the measurement of the total zinc concentration [68]. Dale et al. [65] validated AgNPs’ distribution and silver speciation in freshwater sediments predicted by a simple one-dimensional diagenetic model, as well as calibrated the model using data collected from the mesocosm scale. There are few studies about the validation of EFMs for ENMs in mesocosm-scale studies, because accurate descriptions of fate processes at the mesocosm-scale are often challenging owing to the predominance of highly heterogeneous conditions [118,119]. Despite the validation of other model outcomes or input parameters, EFMs should further be validated at the mesocosm-scale by comparing PECs to the measurement of ENMs.

This review covers three types of existing exposure models for ENMs that have varied complexity and spatial and temporal resolutions. In regulatory applicability, the multimedia box models (i.e., the SimpleBox) have been indicated as the first-tier model in the European Chemical Agency’s (ECHA’s) guidance document in exposure assessment of chemicals [60]. Given the similarity, the SB4N and other related box models are likely directly applicable in the regulatory context for ENMs. The MFAMs has been suggested to be compatible with the material flow concept adopted by ECHA and would likely be applicable [28]. The site-specific models, such as WASP8, describe in greater detail the local environment, such as hydrology, and river cross-section with temporal consideration and is traditionally used to model nutrients and other pollutants in water quality management [120]. This model seems to align with the high-tier models indicated by the ECHA document that can be used when the refinement of chemical risk assessment is warranted [60].

## 5. Summary

The recent development of EFMs and the understanding of emerging ENM fate processes were comprehensively reviewed. We found that ENM fate processes, such as heteroaggregation and dissolution, are the most common ones that are considered in recent EFMs. With the strong advances in emerging ENM fate processes, such as photoreaction and sulfidation, future EFMs should begin to incorporate and evaluate the relative importance of these emerging processes. Sensitivity analysis can be a useful tool to evaluate the relative importance of ENM fate processes in EFMs that comprise multiple concurrent fate and hydrological processes. The consideration of weathered/transformed ENMs, including ENMs released from products that are embedded in product matrices, also represent an important aspect in ENMs’ modeling, as these transformed ENMs often have fate and toxic behaviors distinct from parent ENMs. Recent advances in surface water fate and transport models for ENMs have significantly enhanced our ability in the aspects mentioned above. Opportunities exist in using experimentally derived fate parameters and by coupling MFAMs (i.e., ENM environmental release) and EFMs to improve the accuracy of PECs’ prediction. Nevertheless, further development of EFMs for ENMs especially regarding model validation is urgently needed.
